# Supplementary material for: FastqPuri: high-performance preprocessing of RNA-seq data
Source: BMC Bioinformatics. 2019 May 3;20:226. doi: 10.1186/s12859-019-2799-0 (PMC6500068; doi:10.1186/s12859-019-2799-0)
Supplement: Supplementary file 2 — Archive of FastqPuri. Archive containing all files needed to install and run FastqPuri v1.0.6. Date stamp March 22, 2019. (GZ 47,819 kb) [file 12859_2019_2799_MOESM2_ESM.gz › FastqPuri-1.0.6/html/globals_func.html]

FastqPuri: File Members


FastqPuri


### - a -

- align\_uint32()
  : trim.c
- align\_uint64()
  : trim.c
- alignDS\_uint64()
  : trimDS.c

### - b -

- belongsto()
  : stats\_info.c
- buffer\_output()
  : io\_trimFilter.c
  , io\_trimFilter.h
- buffer\_outputDS()
  : io\_trimFilterDS.h

### - c -

- catExec()
  : fopen\_gen.c
- check\_path()
  : tree.c
  , tree.h
- cmpfunc()
  : stats\_info.c
- command\_Qreport()
  : Rcommand\_Qreport.c
  , Rcommand\_Qreport.h
- command\_Sreport()
  : Rcommand\_Sreport.h
  , Rcommand\_Sreport.c
- compact\_kmer()
  : bloom.c
  , bloom.h
- compress()
  : fopen\_gen.c
- contains()
  : bloom.h
  , bloom.c
- count\_char()
  : str\_manip.h
  , str\_manip.c
- create\_Bfilter()
  : bloom.h
  , bloom.c

### - f -

- fcompress()
  : fopen\_gen.c
- fopen\_gen()
  : fopen\_gen.c
  , fopen\_gen.h
- free\_all\_nodes()
  : tree.c
  , tree.h
- free\_Bfilter()
  : bloom.c
  , bloom.h
- free\_Bfkmer()
  : bloom.c
  , bloom.h
- free\_fasta()
  : fa\_read.c
  , fa\_read.h
- free\_info()
  : stats\_info.h
  , stats\_info.c
- free\_parTF()
  : struct\_trimFilter.h
  , struct\_trimFilter.c
- funcompress()
  : fopen\_gen.c

### - g -

- get\_first\_tile()
  : stats\_info.c
  , stats\_info.h
- get\_fqread()
  : fq\_read.h
  , fq\_read.c
- get\_new\_pool()
  : tree.c
  , tree.h
- get\_tile\_lane()
  : stats\_info.c
- getarg\_makeBloom()
  : init\_makeBloom.c
  , init\_makeBloom.h
- getarg\_makeTree()
  : init\_makeTree.c
  , init\_makeTree.h
- getarg\_Qreport()
  : init\_Qreport.c
  , init\_Qreport.h
- getarg\_Sreport()
  : init\_Sreport.c
  , init\_Sreport.h
- getarg\_trimFilter()
  : init\_trimFilter.h
  , init\_trimFilter.c
- getarg\_trimFilterDS()
  : init\_trimFilterDS.h
  , init\_trimFilterDS.c

### - i -

- ignore\_line()
  : fa\_read.c
- init\_alLUTs()
  : adapters.c
  , adapters.h
- init\_Bfilter()
  : bloom.c
  , bloom.h
- init\_Bfkmer()
  : bloom.c
  , bloom.h
- init\_DSadap()
  : trimDS.c
  , trimDS.h
- init\_entries()
  : fa\_read.c
- init\_fa()
  : fa\_read.c
- init\_info()
  : stats\_info.c
  , stats\_info.h
- init\_LUTs()
  : bloom.c
  , bloom.h
- init\_map()
  : Lmer.c
  , Lmer.h
- insert\_and\_fetch()
  : bloom.c
  , bloom.h
- insert\_entry()
  : tree.h
  , tree.c
- insert\_Lmer()
  : tree.h
  , tree.c
- is\_read\_inBloom()
  : trim.h
  , trim.c
- is\_read\_inTree()
  : trim.h
  , trim.c

### - l -

- Lmer\_sLmer()
  : Lmer.c
  , Lmer.h

### - m -

- main()
  : makeBloom.c
  , makeTree.c
  , trimFilterDS.c
  , Qreport.c
  , trimFilter.c
  , Sreport.c
- multiHash()
  : bloom.h
  , bloom.c

### - n -

- new\_node\_buf()
  : tree.c
  , tree.h
- Nfree\_Lmer()
  : trim.c
- nkmers()
  : fa\_read.c
  , fa\_read.h
- no\_lowQ()
  : trim.c
- no\_N()
  : trim.c
- Ntrim\_ends()
  : trim.c

### - o -

- obtain\_score()
  : adapters.c
  , adapters.h
- obtain\_scoreDS()
  : trimDS.c

### - p -

- pack\_adapter()
  : adapters.c
  , adapters.h
- pack\_reads()
  : trimDS.c
- print\_info()
  : stats\_info.c
  , stats\_info.h
- printHelpDialog\_makeBloom()
  : init\_makeBloom.c
  , init\_makeBloom.h
- printHelpDialog\_makeTree()
  : init\_makeTree.c
  , init\_makeTree.h
- printHelpDialog\_Qreport()
  : init\_Qreport.c
  , init\_Qreport.h
- printHelpDialog\_Sreport()
  : init\_Sreport.c
  , init\_Sreport.h
- printHelpDialog\_trimFilter()
  : init\_trimFilter.c
  , init\_trimFilter.h
- printHelpDialog\_trimFilterDS()
  : init\_trimFilterDS.h
  , init\_trimFilterDS.c
- process\_seq()
  : adapters.h
  , adapters.c

### - q -

- Qtrim\_ends()
  : trim.c
- Qtrim\_frac()
  : trim.c
- Qtrim\_global()
  : trim.h
  , trim.c
- QtrimDS()
  : trimDS.c

### - r -

- read\_Bfilter()
  : bloom.c
  , bloom.h
- read\_fasta()
  : fa\_read.h
  , fa\_read.c
- read\_info()
  : stats\_info.c
  , stats\_info.h
- read\_tree()
  : tree.h
  , tree.c
- realloc\_fa()
  : fa\_read.c
- resize\_info()
  : stats\_info.c
  , stats\_info.h
- rev\_comp()
  : Lmer.h
  , Lmer.c

### - s -

- save\_Bfilter()
  : bloom.c
  , bloom.h
- save\_tree()
  : tree.h
  , tree.c
- size\_fasta()
  : fa\_read.c
  , fa\_read.h
- str\_isascii()
  : str\_manip.h
  , str\_manip.c
- strindex()
  : str\_manip.c
  , str\_manip.h
- strindexC()
  : str\_manip.c
- string\_seq()
  : fq\_read.h
  , fq\_read.c
- strsplit()
  : str\_manip.h
  , str\_manip.c
- sweep\_fa()
  : fa\_read.c

### - t -

- tree\_from\_fasta()
  : tree.c
  , tree.h
- trim\_adapter()
  : trim.h
  , trim.c
- trim\_adapterDS()
  : trimDS.c
  , trimDS.h
- trim\_sequenceN()
  : trim.c
  , trim.h
- trim\_sequenceQ()
  : trim.h
  , trim.c

### - u -

- uncompress()
  : fopen\_gen.c
- update\_ACGT\_counts()
  : stats\_info.c
  , stats\_info.h
- update\_ACGT\_pos()
  : stats\_info.c
  , stats\_info.h
- update\_info()
  : stats\_info.h
  , stats\_info.c
- update\_QPosTile\_table()
  : stats\_info.h
  , stats\_info.c

### - w -

- write\_info()
  : stats\_info.c
  , stats\_info.h
- write\_summary\_TF()
  : io\_trimFilter.h
  , io\_trimFilter.c
- write\_summary\_TFDS()
  : io\_trimFilterDS.h


---

Generated on Mon Mar 19 2018 23:42:01 for FastqPuri by  

 1.8.14
